# Southern Surgical and Gynecological Association

**Published:** 1893-01

**Authors:** 


### *SOCIETY REPORTS.*

---

### SOUTHERN SURGICAL AND GYNECOLOGICAL ASSOCIATION.

[CONTINUED.]

### SECOND DAY—AFTERNOON SESSION.

The Association was called to order at 2:30 P. M., with the First Vice-President, Dr. C. Kollock, in the chair.

The President delivered his Annual Address. He took for his subject,

COMPATIBILITY OF CONSERVATIVE AND AGGRESSIVE SURGERY.

Published in December JOURNAL.

DR. AP. MORGAN VANCE, of Louisville, read a paper entitled

A PLEA FOR MORE RAPID SURGICAL WORK, in which he said a great number of surgeons pay little attention to the time consumed in an operation, or to the nicety of manipulation and dextrous use of instruments that our forefathers prided themselves upon. He had seen on numerous occasions the work of our most distinguished surgeons, and seen deaths occur from prolonged anæsthesia and too much time consumed in operation which would not have taken place if much unnecessary time had not been wasted. The habit of starting the anæsthetic before all preparations were completed was very reprehensible.

The paper was discussed by Drs. Arch. Dixon, Rufus B. Hall, Geo. A. Baxter and Edwin Ricketts, all endorsing the position taken by the essayist.

DR. CHARLES A. L. REED, of Cincinnati, contributed a paper entitled

SURGERY OF THE URETERS WITH REPORT OF CASES.

He said that surgery of the ureter is one of the developmental subjects of abdominal surgery. These out-of-the-way conduits exercising functions that are vital in character were liable to diseased conditions which baffle the resources of the diagnostician and tax the ingenuity of the operator. For purposes of diagnosis the physical means at our disposal may be briefly summarized as follows: (1) Exploration of the lower end of the ureter by digital examination, (a) through the vagina, (b) the rectum, and (c) the bladder; (2) exploration of the lower end of the ureters by the sound passed through the urethra and bladder into the ureters; (3) exploration of the central portion of the ureters by abdominal lumbar palpation—an expedient of practical value only in cases of extreme ureteral distension occurring in very thin subjects; (4) exploration of the upper end of the ureters by exploratory nephrotomy. Each of these several expedients might be amplified, but it would be uncalled for in the presence of such distinguished members. Dr. Reed said that since catheterization of the ureters has been popularized in this country chiefly through Kelly, and since the technique of the procedure has become understood by those who have studied it, the diagnosis of disease within and surrounding these tubes is vastly more common. The digital exploration of that portion of the ureters lying within easy reach from the vagina or rectum is readily practiced by those who have carefully studied the anatomy of the parts. Digital exploration through the urethra and bladder is an easy expedient so far as the surgeon is concerned and often leads to the elucidation of important pathological facts, but the speaker is forced to believe that it is not without danger to the patient. He has been forced into this belief by one case of incontinence lasting for nearly a year and by two cases of weakened power of retention, one of which is now of quite two years standing. Dr. Reed said that abdominal section for diagnosis of ureteral conditions, notably in cases of suspected calculus, is entirely justifiable. He then reported a case of periureteritis; stricture; kolpo-cysto-ureterotomy, with recovery. The second case was one of cicatricial stricture of an excised ureter; hydro-nephrosis; with recovery. The third case, one of pyo-nephrosis; nephrectomy; remaining urethral disease.

DR. WILLIAM WARREN, of Buffalo, read a paper entitled

### SPECIALISM IN MEDICINE PARTICULARLY AS RELATED TO SURGERY AND GYNECOLOGY.

He summarized his argument thus :

1. There is essential need for specialists. Divisions of labor in every field are demanded, and nowhere more than in medicine.

2. Specialists being a necessity, they must equip themselves by years of study, and devote themselves to a still greater number of years of general practice before they are justified in offering themselves as specialtists.

3. They must conduct themselves in such a way as to merit the respect of the general practitioner, and to invite his co-operation in their work.

4. The unwritten ethics of specialism demand that there shall be reciprocal relationship maintained not only among specialists themselves, but also between specialist and general practitioners.

5. The opportunities for perfection in special lines of medical study are so great, and medical literature in both journalistic and text-book form is so rich, that an awful responsibility is entailed.

6. The schools ought to discourage any and all students who give promise of entering upon the practice of a specialty as soon as the college doors are passed, and before the swaddling clothes of the professional tyro are slipped.

DR. R. M. CUNNINGHAM, of Birmingham, Ala., followed with a paper entitled

### THE GENERAL PRACTITIONER AS A GYNECOLOGIST.

He said the general practitioner should not undertake work that can be better and more safely done by the specialist, provided one is obtainable. He should be willing to do and attempt the most radical and dangerous operations when necessary to save life, provided a specialist or one better prepared to do the work cannot be obtained. In cases not necessarily dangerous, or in which life does not become more or less a burden, but in which a cure can be effected only by a radical procedure, but which may be materially benefited, or symptomatically relieved by milder methods, he should adopt the latter and not the former. In many cases the field is clearly his own, belongs to him, and he should be prepared and competent to treat them with safety and success.

DR. W. F. WESTMORELAND of Atlanta, read a paper on

### SPECIALISM IN MEDICINE.

He said there were two kinds of specialists, the one with his preconceived ideas, which become warped, who always suffers from astigmatism, etc., who is a graduate of his kind. The other kind was the man who has worked his way by his generally acknowledged ability in any particular line.

DR. HOWARD A. KELLY, of Baltimore, read a paper entitled

### A PRELIMINARY REPORT ON THE MORPHOLOGY OF OVARIAN AND MYOMATOUS TUMORS.

The speaker said the form of abdomen characteristic of large ovarian cysts is a globular or ovoid distension of a part or the whole of the abdominal wall, pushing out the infra-umbilical portion much more than the supra-umbilical, at least so long as the tumor occupies the lower half or two-thirds of the abdomen. This enlargement is uniform in parovarian cysts and polycystic tumors, exhibiting but few bosses, due to the fact that the latter are composed of one or two large cysts associated with a mass of smaller ones, and the large cyst is best accommodated in the median line in the distended concave anterior abdominal wall, while the smaller ones at the side or back consequently do not show. Prominent exceptions to the general rule just enunciated that pelvic tumors distend most markedly the inferior abdominal zone are the notable stretching of the upper abdomen in very fat women with large ovarian tumors, and the like distension in rachitic dwarfs in advanced pregnancy. Nodular myomata, on the other hand, stand out in marked contrast to the smooth outlines of cystic tumors in giving to the lower abdomen a lumpy bossed appearance, thus exhibiting through muscles and skin a softened exaggeration of their irregular outlines. This peculiarity still remains prominent although softened, after these tumors have undergone fibrocystic degeneration.

Cystic tumors filling the pelvis and part of the abdomen are but rarely found to originate in some upper abdominal tumor. The speaker presented for demonstration a photograph of an enormous kidney, containing over a gallon of pus, extending from the pelvis floor up through the abdomen and pushing up the left ribs.

### THIRD DAY—MORNING SESSION.

DR. A. M. CARTLEDGE, of Louisville, read a paper entitled

### THE PRESENT STATUS OF DRAINAGE IN SURGERY.

He presented the following summary:

1. The principle of artificial drainage in surgery, while very ancient, was imperfectly understood, and was oftentimes as much a factor for evil as for good.

2. Though our knowledge of the principles which govern a healthy regeneration of wounded structures has greatly advanced, and our progress in wound therapeutics kept pace, we fail to appreciate how artificial drainage can be altogether dispensed with in surgical practice.

3. To lessen the use of artificial drainage it is necessary to thoroughly apply the principles of asepsis and antisepsis, combined with buried sutures, fixation and alimentary or systemic drainage.

4. Where from any reason the production of a serum cannot be controlled, its removal by drainage is a safer surgical measure than any attempt at sterilization *in situ.*

5. The time required for primary drainage is from twenty-four to sixty hours; to wait longer is to encourage trouble, to remove sooner than twenty-four hours is taking risks not warranted in the premises.

6. Capillary is to be preferred to tubular drainage in wounds other than those of the large cavities. For this purpose absorbent material should be selected, catgut being the best.

7. Where it is desirable to combine hemostasis and drainage in the same measure, the strips of iodoform gauze, as recommended by Mikulicz, fulfill a most useful purpose.

8. Where natural drainage can be utilized without producing unsightly cicatrices, artificial drainage should be dispensed with; when feasible combine the two.

9. Wounds involving the brain and cord had best be drained to avoid mechanical violence to the function of delicate structures by retained serum.

10. Necessity for artificial drainage will most often arise in wounds invading the large cavities; here inflexible tubular drains (glass) best meet the requirements, aided or not by materials acting by capillarity.

11. The method of secondary suture after primary wound secretion is over, advised by Kocher, seems to possess no advantage over drains that have to be removed, and certainly is not to be compared in convenience, comfort, etc., to the patient, to absorbable capillary drains.

DR. WILLIAM H. MYERS, of Fort Wayne, Indiana, read a paper on

### THE TREATMENT OF TUBERCULAR PERITONITIS.

He said when we have arrived at the conclusion that peritonitis is present, and have discovered the cause, the blow must be struck simultaneously with the onset. No delay can safely be tolerated, the only hope of rescue being the sudden arrest of the disease. By the time that the normal outlines of the abdomen are obscured by tympanitic distensions, respiration quickened and shallow, the pulse rapid and wiry, the supreme moment for precise diagnosis is passed. Abdominal section for tubercular peritonitis was the most recent triumph of surgery. Dr. Myers had treated three cases of tubercular peritonitis by abdominal section, washing out the abdominal cavity and drainage, with complete recovery.

DR. G. FRANK LYDSTON, of Chicago, followed with a paper entitled

BACTERIOLOGICAL RESEARCH IN ITS RELATIONS TO THE SURGERY OF THE GENITO-URINARY ORGANS.

The author said that in his opinion modern bacteriological and pathological research has nowhere been more productive of scientific and practical progress than in the special field of genito-urinary surgery. He would not attempt to decide the question as to whether under certain circumstances the microbial organisms which are constantly to be found in the secretions of the genito-urinary tract, are causal factors in pathogenesis of various forms; or, on the other hand, to decide the precise relation of heterogenetic organisms to the same morbid process. The relation between what may be termed the normal germ and germs of non-pathogenic properties must certainly be left to the practical microbiologist. We are warranted, however, in drawing certain inferences and making certain practical deductions from what we know of the evolutionary laws of progression, differentiation and adaptation to environment. Many of the diverse forms of disease of microbial origin were doubtless embraced under the omnibus term of "urinary infection." The present state of our knowledge does not admit of arbitrary differentiation between them. It is sufficient to say that many forms of organic and functional change affecting the genito-urinary tract are of microbial origin. These processes range in severity from a general infection with effusion and perhaps suppuration in joint cavities to so simple a local lesion as a chronic prostatic irritation. The author quoted the researches of such modern authors as Reginald Harrison, Halle, Rovsing, Krögius, Bumm, Albarran and Guyon.

DR. JOSEPH TABER JOHNSON, of Washington, D. C., read a paper entitled

OVARIOTOMY IN OLD WOMEN, in which he reported three cases, and felt quite sure that prolonged anæsthesia and manipulation within the peritoneal cavity would have proved fatal. The first patient was sixty-seven years of age, and the tumor removed weighed fifty-two pounds. The second case was one of multilocular ovarian tumor. The patient was sixty-eight years of age, and the tumor weighed sixty-four pounds. On October 10th of this year he removed an ovarian tumor weighing fifty-six pounds from a lady who was sixty-seven years old, but who looked to be one hundred. Improved methods, quicker operations, antiseptic technique and provisions against shock show thirty-eight recent cases between the ages of sixty-seven and eighty-two, with only two deaths against twenty-four cases, done twenty years ago, between the ages of sixty and sixty-seven with a record of six deaths. These figures demonstrate, in addition to improved technique, the surprising fact that old age is no contraindication against ovariotomy.

DR. BEDFORD BROWN, of Alexandria, Va., read a paper entitled

THE SIMPLE, SEPTIC, TRAUMATIC AND SPECIFIC FORMS OF CERVICITIS AND THEIR TREATMENT.

Simple cervicitis arises alone from simple causes. It never originates from infection of any kind. It could exist for an indefinite period without infecting surrounding structures. For many years the author, in the treatment of this affection, has addressed his remedies to the interior of the cervical canal alone, whether he used nitrate of silver, sulphate of copper, carbolic acid or iodine. Septic cervicitis arises always from septic infection and in itself becomes a center of septic infection for the pelvic structures connected by lymphatic communication. Contact with the os of portions of putrescent placenta, membranes, coagula or septic discharges from diseased uteri were the common causes. Antiseptic measures alone could counteract septic infection and inflammation, whether in the form of septicæmic fever or local inflammatory action. All other agencies were simply palliative or adjuvant in character. Traumatic cervicitis was simply inflammation and congestion of the cervix from wounds inflicted on that body either during labor, abortion or from the use of dilating instruments. The author treats this form of cervicitis by means of a solution of nitrate of silver, varying in strength from a scruple to half a drachm applied in the canal and over the entire cervix. He finds that most of his cases of open and all cases of concealed wounds heal by this method. Specific cervicitis may arise either from gonorrhœal or syphilitic infection. In the early stages he resorts to douches containing peroxide of hydrogen in the proportion of one part to three-fourths of boiled water, and also permanganate of potash, one grain to the ounce of water.

DR. JAMES EVANS, of Florence, S. C., contributed a paper on

### SHOCK.

The speaker said in the severe injuries inflicted on the body by accident, and in the major operations of surgery, not the least element of danger to life is the condition known as shock which rapidly supervenes. The degree of shock is not determined solely by the extent and gravity of the physical injury. Certain idiosyncrasies of constitution, the character of the force which inflicted the injury, and the circumstances under which it occurred, are potent factors in its determination. Individuals of a highly wrought and exquisitely nervous organization bear pain with far less fortitude and are more susceptible to shock than those of dull and obtuse intellects and blunted sensibilities. The author reported a case in point. In laying the foundation of a bridge across the Pee Dee river in South Carolina, an immense block of granite weighing over a ton was being lowered into a pit forty-four feet in depth, at the bottom of which was a man who was to direct when it was in proper position. When this huge block of stone was suspended over the pit the cable holding it began to slip, and the man below was warned to crouch in a corner, as it would inevitably fall. The rock did fall, and the man in the pit miraculously escaped without injury, but he was taken out in a perfectly lifeless condition and was exceedingly ill for more than a week.

A MANIPULATIVE MISTAKE AND ITS CONSEQUENCES.

This paper was read by Dr. George Ross, of Richmond, Va. The author related the case of a woman who had suffered from unremitting, agonizing tenesmus, the result of a mass which she carried for seven years in her bladder, and which proved to be on inspection a pledget of absorbent cotton once saturated with iodine, in shape a truncated cone, and thinly incrusted with phosphate of lime. The patient believed it was introduced by her first physician, who, when attempting to apply an intra-uterine dressing, mistook the urethra for the cervical canal.

DR. WILLIAM PERRIN NICOLSON, of Atlanta, made some remarks on

HARELIP OPERATIONS, in which he advocated the use of a simple suture instead of a pin, and also recommended paring the edges.

DR. EDWIN RICKETTS, of Cincinnati, read a short paper entitled

CHOLECYSTOTOMY, WITH THE REPORT OF A CASE.

He had operated thirteen times for obstruction of the gall ducts. The patient, a lady, thirty-four years of age, married, consulted him last June. She had never suffered markedly from jaundice, nor from acute attacks of hepatic colic; no marked distention over the region of the gall-bladder; abdominal wall at least three inches in thickness; some general tenderness of the liver elicited by percussion. The patient had the characteristic putty-colored stools, and was losing flesh rapidly. The author advocated allowing a glass drainage tube to remain in until the common duct was opened, and then, if necessary to make an anastomosis between the gall-bladder and the duodenum.

The following officers were elected:

*President*—Dr. Bedford Brown of Alexandria, Va.

*First Vice-President*—Dr. Jos. Price of Philadelphia.

*Second Vice-President*—Dr. Geo. A. Baxter of Chattanooga.

*Secretary*—Dr. W. E. B. Davis of Birmingham, Ala.

*Treasurer*—Dr. Hardin P. Cochrane of Birmingham.

*Place of meeting*—New Orleans, La. Time, second Tuesday in November, 1893.

*Chairman of Committee of Arrangements*—Dr. Albert Miles.

